# Evaluation and comparison of voided urine and bladder washing cytologies according to the Paris system

**DOI:** 10.1590/1806-9282.20251936

**Published:** 2026-06-29

**Authors:** Asiye Şafak Bulut, Yalçın Kızılkan

**Affiliations:** 1 Bilkent Şehir Hastanesi, Department of Pathology – Ankara, Turkiye.; 2 Bilkent Şehir Hastanesi, Department of Urology – Ankara, Turkiye.

**Keywords:** Urine, Urinary bladder neoplasms, Cytodiagnosis

## Abstract

**OBJECTIVE:**

Cystoscopy, biopsy, and urine cytology are gold standards for the diagnosis and surveillance of bladder carcinoma. Bladder washings, obtained via cystoscopy or by catheterization, are common in routine cytology practice, whereas voided urine is evaluated less frequently. The aim of this study was to perform a comparative cytologic assessment of voided urine and bladder washing specimens in patients with bladder carcinoma.

**METHODS:**

In this prospective study, second-morning voided urine and bladder washing samples were obtained from 75 patients with histologically confirmed bladder carcinoma over a 3-year period. Samples were re-evaluated cytologically according to the Paris system.

**RESULTS:**

Bladder washing cytology showed higher sensitivity (81 vs. 62%) and the correlation coefficient (0.617 vs. 0.487), compared to voided urine cytology.

**CONCLUSION:**

While bladder washing cytology showed superior diagnostic performance with a higher area under the curve and sensitivity in this cohort, voided urine cytology remains a valuable non-invasive alternative despite its lower sensitivity.

## INTRODUCTION

Urine cytology plays a pivotal role in the detection and follow-up of urothelial carcinomas. The Paris system for reporting Urinary Cytology (TPS), a standardized reporting system with an emphasis on the detection of high-grade urothelial carcinoma, has been widely used since its introduction in 2016^1^. It was revised in 2022 (TPS 2.0), with clarification of existing diagnostic categories^2^.

The primary objective of this study was to perform a comparative cytologic evaluation of voided urine and bladder washing specimens based on TPS 2.0 criteria.

## METHODS

A prospective study was conducted to compare the diagnostic performances of voided urine and bladder washing cytologies. A total of 75 patients who underwent cystoscopic tumor resection for either newly diagnosed or recurrent tumors between 2019 and 2021 were included. Patients with primary bladder malignancies other than the urothelial carcinoma, metastatic carcinomas, and non-epithelial tumors were excluded.

Clinical data, including age, gender, medical history, and histopathology reports, were retrieved from patient records. Voided urine specimens were collected as second morning voids. Bladder washing samples were obtained either prior to or during flexible cystoscopy.

Cytological specimen volumes were recorded. Samples prepared by the cytospin technique (Thermo Scientific, Pittsburgh, Pennsylvania) were stained with both May-Grünwald-Giemsa and Papanicolaou stains. All slides evaluated according to the Paris system were then re-evaluated and classified according to the revised version, TPS 2.0.

Bladder biopsy specimens were obtained following bladder washings. Routine histopathological protocols were applied to tissues. Pathological parameters were recorded from pathology reports.

Statistical analysis for distributions, comparisons, correlations, and diagnostic performances was performed using SPSS software, version 22.0 (IBM Corp., Armonk, NY, USA).

## RESULTS

A total of 150 cytological specimens, comprising voided urine and bladder washings from 75 patients, were analyzed. There were 65 males and 10 females. The age range was 43–91 years (mean: 71.3) for males and 48–87 years (mean: 73.4) for females.

Among 75 patients, 54 had past or concomitant co-morbidities like malignancy, diabetes, hypertension, coronary artery disease, lung disease, or rheumatologic disease.

Primary tumors (n=55) presented predominantly with painless hematuria, whereas recurrent tumors (n=20) were generally identified during routine follow-up. Tumors were low grade in 38 and high grade in 37 patients according to the World Health Organization/International Society of Urological Pathology classification^3^.

Cytological sample volumes were 3–60 mL (mean: 17.89 mL) for voided urine and 5–30 mL (mean: 16.71 mL) for bladder washings. Sample volume didn't significantly affect cytologic diagnosis (p>0.05). However, hypocellularity and suboptimal preparations observed in 15 urine and 8 bladder washings caused significantly lower categorization compared to non-hypocellular ones, both for voided urine cytology (VUC) (p=0.002) and in bladder washing cytology (BWC) (p=0.0017).

Acute inflammation was observed in 18 patients (24%). High-grade carcinomas were more frequently associated with inflammatory samples compared to non-inflammatory ones (67 vs. 44%).

The cytological-histopathological correlations for VUC and BWC were shown in Tables 1 and 2. Lymphovascular invasion was present in 3 cases (4%), and tumor necrosis was observed in 19 cases (25%). Risk of high-grade malignancy (ROHM) was 0% for non-diagnostic (ND), 0% for Negative for High-Grade Urothelial Carcinoma (NHGUC), 35% for Atypical Urothelial Cells (AUC), 67% for Suspicious for High-Grade Urothelial Carcinoma (SHGUC), and 83% for High-Grade Urothelial Carcinoma (HGUC) for VUC, and 50, 0, 18, 93, and 86% for BWC, respectively.

**Table 1:** Cytological-histological correlation for voided urine cytology.

Histopathology	Voided urine cytology					
	ND n (%)	NHGUC n (%)	AUC n (%)	SHGUC n (%)	HGUC n (%)	Total
Ta LG	–	3 (4)	23 (31)	4 (5)	3 (4)	33 (44)
Ta HG	–	–	7 (9)	4 (5)	3 (4)	14 (18)
T1 LG	1 (1)	1 (1)	3 (4)	–	–	5 (6)
T1 HG	–	–	6 (8)	4 (5)	9 (12)	19 (25)
T2 HG	–	–	1 (1)	–	3 (4)	4 (5)
Total	1 (1)	4 (5)	40 (53)	12 (15)	18 (24)	75 (100)

LG: low-grade; HG: high-grade ND: non-diagnostic; NHGUC: negative for high-grade urothelial carcinoma; AUC: atypical urothelial cells; SHGUC: suspicious for high-grade urothelial carcinoma; HGUC: high-grade urothelial carcinoma.

**Table 2:** Cytological-histological correlation for bladder wash cytology.

Histopathology	Bladder wash cytology					
	ND n (%)	NHGUC n (%)	AUC n (%)	SHGUC n (%)	HGUC n (%)	Total
Ta LG	–	4 (5)	23 (31)	3 (4)	3 (4)	33 (44)
Ta HG	–	–	4 (5)	6 (8)	4 (5)	14 (18)
T1 LG	1 (1)	–	4 (5)	–	–	5 (6)
T1 HG	1 (1)	–	1 (1)	5 (7)	12 (16)	19 (25)
T2 HG	–	–	1 (1)	–	3 (4)	4 (5)
Total	2 (3)	4 (5)	33 (44)	14 (19)	22 (29)	75 (100)

LG: low-grade; HG: high-grade; ND: non-diagnostic; NHGUC: negative for high-grade urothelial carcinoma; AUC: atypical urothelial cells; SHGUC: suspicious for high-grade urothelial carcinoma; HGUC: high-grade urothelial carcinoma.

In statistical analysis, the Kolmogorov-Smirnov test showed that the cytological categories for both voided urine and bladder washings did not follow a normal distribution (p<0.001).

There was a significant positive correlation between age and BWC results (rho=0.240, p=0.038), and older patients presented with higher cytological atypia. Muscle invasion showed a positive correlation with higher cytological categories, while acute inflammation and hypocellularity did not impact the diagnostic accuracy in this cohort.

The 2×2 contingency tables comparing VUC and BWC are presented in Table 3. In accordance with the Paris system criteria, "positive" cytology was defined as SHGUC and HGUC, while high-grade histopathology served as the gold standard. Correlations and diagnostic performances were evaluated using Spearman's rank correlation and receiver operating characteristic curves. Both sampling methods showed a statistically significant correlation with histopathology (p<0.001); however, BWC demonstrated a stronger relationship (rho: 0.617 vs. 0.487). Furthermore, BWC exhibited higher sensitivity (81 vs. 62%) and superior overall diagnostic performance (AUC 0.835 vs. 0.756) in detecting high-grade lesions. As the study focused on differentiation between low-grade and high-grade tumors and lacked a benign control group, specificity and positive and negative predictive values could not be evaluated. Consistency between the two sampling methods were evaluated using the Weighted Kappa and the Wilcoxon Signed-rank test, demonstrating substantial agreement (κ: 0.787) and no statistically significant difference in Paris category distributions (p=0.180), indicating that the two methods are clinically comparable.

**Table 3:** 2×2 contingency table for voided urine cytology and bladder washing cytology.

Cytology	Voided urine			Bladder washing		
	High-grade pathology	Low-grade pathology	Total	High-grade pathology	Low-grade pathology	Total
SHGUC, HGUC	23 (TP)	7 (FP)	30	30 (TP)	6 (FP)	36
ND, NHGUC, AUC	14 (FN)	31 (TN)	45	7 (FN)	32 (TN)	39
Total	37	38	75	37	38	75

TP: true positive; FP: false positive; FN: false negative; TN: true negative. ND: non-diagnostic; NHGUC: negative for high-grade urothelial carcinoma; AUC: atypical urothelial cells; SHGUC: suspicious for high-grade urothelial carcinoma; HGUC: high-grade urothelial carcinoma.

## DISCUSSION

Urinary cytology serves as a crucial adjunct to cystoscopy in the evaluation of urothelial lesions. TPS, accepted by over 90% of institutions, is user-friendly and particularly effective in detecting HGUC in urine cytology^4^. It was developed mainly for ThinPrep and SurePath preparations, but it is also applicable to conventional smears and cytospin techniques. In a comparative study, cytospin, ThinPrep, and conventional methods evaluated on bladder washings from 31 patients demonstrated strong concordance with histopathological findings, in which cytospin yielded 100% specificity, 81.5% sensitivity, and 83.9% diagnostic accuracy^5^. In another study, cytospin-prepared urine samples collected by different methods in 303 patients, 86.8% diagnostic accuracy, 94.7% sensitivity, and 73.9% specificity were reported, showing that TPS-defined standards are reliable on cytospin-prepared slides^6^. Our study similarly achieved interpretable smears via the cytospin technique.

In cytologic evaluation, adequacy of urine specimens depends on four features: collection type, cellularity, volume, and cytomorphology. According to TPS 2.0, any specimen showing definitive atypia is considered adequate regardless of other parameters^2^. In a study evaluating cytospin preparations from 162 voided and 152 instrumented urine samples, VUC sensitivity correlated significantly with urothelial cellularity but not with specimen volume. Cellularity and sensitivity were lower in voided specimens compared to instrumented collections^7^. In our study, the sample volume was ≤5 mL in five VUC and one BWC samples; only one of the VUC and two of the BWC samples; were inadequate for diagnosis. Sample volumes didn't significantly affect cytologic categorization. On the other hand, 15 VUC and 8 BWC exhibited low cellularity, which negatively influenced interpretation and led to lower categorization, probably due to the limited cell content. These data supported the hypothesis that neither volume nor cellularity alone reliably predicts adequacy.

An inflammatory background may influence specimen adequacy and cytologic interpretation. In our cohort, acute inflammation was observed in both VUC and BWC samples from 18 patients, six with low-grade tumors and twelve with high-grade tumors. Most of the samples were categorized as AUC, SHGUC, or HGUC, with only two VUC and one BWC reported as benign. Unlike Saarinen et al., who observed a statistically significant downgrading trend in pyuric male specimens, our data did not show any inflammation-related diagnostic shift^8^.

When the frequencies of categories are compared to the reference frequencies in TPS 2.0, AUC, SHGUC, and HGUC are more frequent than NHGUC because our study included only histologically confirmed urothelial carcinoma cases. The frequency of the ND category matched the suggested frequencies^2^.

Bladder washing is the preferred cytologic collection method due to superior cellularity, good preservation of cells, and less contamination in the background. However, voided urine also performs reliably in both diagnosis and surveillance. Kurtycz et al. highlighted high specificity and sensitivity in BWC for HGUC^4^. More recent data from Gómez Cañizo et al. reported high specificity in both positive (92.4%) and suspicious (90.9%) VUC categories, with an overall sensitivity of 65%^9^.

Comparative studies of VUC and BWC are limited. Keller et al. found comparable outcomes in 1,447 patients using non-TPS categorization methods, suggesting flexibility based on local availability^10^. In Sarfaraz et al.'s study, in which the TPS was not used and samples were categorized as negative, suspicious, and positive for high-grade urothelial carcinoma, similarly equivalent performance was reported in voided and instrumented samples^11^. Joudi et al., using TPS, documented higher positivity rates in BWC than VUC during a 6-month follow-up after positive or suspicious cytology; however, voided urine specimens comprised only 13.8% of samples^12^. Planz et al. found no significant specificity and sensitivity differences between voided urine and bladder washings but only one type of specimen obtained from each patient, and none of them provided both types of samples in their study^13^. Struck et al. reported striking specificity (>97%) and moderate sensitivity (∼60%) for both specimens^14^. Conversely, Kumar et al. reported the lowest overall VUC sensitivity (13.33%), cautioning against its use as a screening test^15^. Our results support a correlation between VUC and BWC and align with Gómez Cañizo et al.'s statement that VUC is also suitable for cytologic evaluation due to its high specificity for high-grade tumors^9^.

ROHM in our study concords with published pooled ROHM rates. In a meta-analysis that reviewed 28 TPS (1.0)-based studies, the pooled ROHM was 17.7, 13.4, 38.65, 12.45, 76.89, and 91.79% for ND, NHGUC, AUC, Low-Grade Urothelial Carcinoma (LGUC), SHGUC, and HGUC, respectively^16^. Pastorello et al. and another meta-analysis reported similar ROHMs^17,18^. Our rates for AUC, SHGUC, and HGUC are consistent with these studies; the difference in ND and NHGUC categories reflects our carcinoma-only cohort.

Previous reports of TPS 2.0 performance demonstrate sensitivity ranging from 37.5 to 93.6% and specificity between 73 and 100%^2^. Pastorello et al.'s review similarly reported 40–84.7% sensitivity and 73–100% specificity^17^. Our findings of 62 and 81% sensitivity for VUC and BWC, respectively, fall within the range.

In this study, BWC demonstrated superior diagnostic accuracy compared to VUC for high-grade tumors (AUC 0.835 vs. 0.756). BWC exhibited strong predictive capability, while VUC showed moderate strength. Predictive accuracy increased significantly when the cytologic category reached SHGUC or higher (HGUC). VUC showed lower cellularity compared to BWC. However, it remains a strong candidate for molecular or specific screening assessments. As demonstrated by Martins et al., urine-based collection serves as an effective tool for specialized diagnostic purposes like human papillomavirus genotyping. This indicates that while voided urine may be less cellular, its ease of use and molecular compatibility maintain its clinical significance in broader screening strategies^19^.

Our study is planned just before the COVID-19 pandemic. As access to the hospital was restricted during that period, the study was completed longer than planned, and TPS 2.0, which was introduced in 2022, was used for cytological evaluation. In TPS 2.0, some diagnostic criteria were revised, diagnostic categories were modified, risk of malignancy for each category based on data was updated, and management recommendations were developed. A new chapter focused on upper urinary tract cytology was involved. Adequacy criteria are unchanged from TPS 1.0. Notable updates have been introduced in the NHGUC category. LGUC, which was previously addressed in a standalone chapter, was abolished and merged into the NHGUC category^2^. Recent studies affirm that TPS 2.0 enhances diagnostic accuracy for HGUC^20,21^.

Study limitations include a relatively small sample size due to the decreased patient admittance during the COVID-19 pandemic (2020–2021), focusing only on carcinoma cases, non-homogeneous cytologic category distribution, and inability to calculate specificity, positive, and negative predictive value due to a lack of benign controls.

## CONCLUSION

Our findings demonstrate that BWC provides significantly higher sensitivity and overall diagnostic accuracy for HGUC compared to VUC. While VUC is a viable, low-cost, non-invasive option, its lower sensitivity suggests it should be used cautiously as a stand-alone tool for high-grade carcinoma detection. The Paris System is reproducible with good histopathological correlation, especially in positive or suspicious categories.

## Data Availability

The datasets generated and/or analyzed during the current study are available from the corresponding author upon reasonable request.
